# Hidradenitis Suppurativa in a Patient with Smith-Magenis Syndrome: A Case Report

**DOI:** 10.7759/cureus.4970

**Published:** 2019-06-22

**Authors:** Shanice A McKenzie, Catherine S Ni, Jennifer L Hsiao

**Affiliations:** 1 Dermatology, David Geffen School of Medicine at University of California Los Angeles, Los Angeles, USA

**Keywords:** hidradenitis suppurativa, smith-magenis syndrome, follicular occlusion

## Abstract

Hidradenitis suppurativa (HS) is an inflammatory disorder characterized by recurrent painful nodules and draining sinus tracts with subsequent scarring. HS has been associated with several dermatologic syndromes including the follicular occlusion tetrad, PASH (pyoderma gangrenosum, acne, and suppurativa hidradenitis), PAPASH (pyogenic arthritis, pyoderma gangrenosum, acne, and suppurativa hidradenitis), and SAPHO (synovitis, acne, pustulosis, hyperostosis, and osteitis) syndromes. However, aside from the well-established association between HS and Down Syndrome, it has rarely been reported in association with other multisystem genetic diseases. We report a case of HS in a 22-year-old man with Smith-Magenis syndrome (SMS), a developmental disorder caused by a chromosomal 17 microdeletion. Obesity and a tendency toward follicular occlusion are features of SMS that may have contributed to the development of HS in our patient. Interestingly, the patient also had several other follicular-based skin diseases including erythromelanosis follicularis faciei, keratosis pilaris, and acne keloidalis nuchae. Dermatologic findings in SMS have been characterized in the literature in the past, but to our knowledge, this is the first case of HS reported in a patient with SMS. We also provide a brief literature review of cases of HS occurring in patients with other multisystem genetic diseases.

## Introduction

Hidradenitis suppurativa (HS) is a chronic relapsing inflammatory disease, predominantly affecting intertriginous areas of the body. Clinically, HS is characterized by painful subcutaneous nodules and abscesses that rupture, fistulize, and scar [1]. In addition to the well-established association between HS and Down Syndrome [2], there have been rare reports of HS in association with other multisystem genetic diseases, including Familial Mediterranean Fever (FMF), keratitis-ichthyosis-deafness syndrome (KID syndrome), Bazex-Dupre-Christol syndrome, and myotonic dystrophy [3-4]. We present what we believe to be the first reported case of HS in a patient with Smith-Magenis syndrome (SMS) and a review of the literature for HS in association with other genetic diseases.

## Case presentation

A 22-year-old man with a history of SMS presented to the dermatology clinic for evaluation of tender cystic lesions in the groin. History was mostly obtained from the patient’s mother, as the patient is developmentally delayed. The patient first started getting HS lesions around age 9, with flares occurring about 4-5 times per year. For the past month, he had been treated with doxycycline 100 mg orally twice daily and topical clindamycin 1% solution twice daily with improvement. Prior to this, he had never received treatment for his skin condition.

The patient’s weight was 275 lbs (BMI 39.3). His other medications included sertraline 100 mg daily and quetiapine 50 mg daily. He denied smoking, alcohol use, or drug use. His aunt may have symptoms of HS, but she has never been diagnosed.

**Figure 1:**
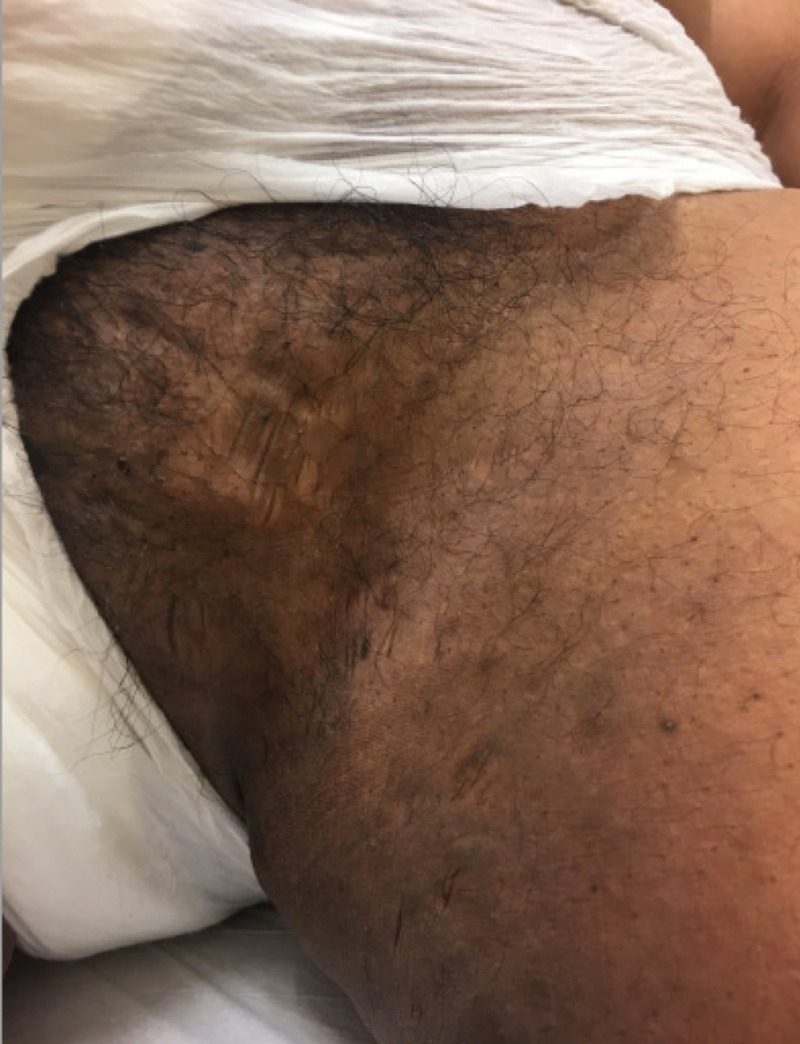
Hidradenitis suppurativa manifesting as hyperpigmented scars and nodules on the groin fold and inner thigh

**Figure 2:**
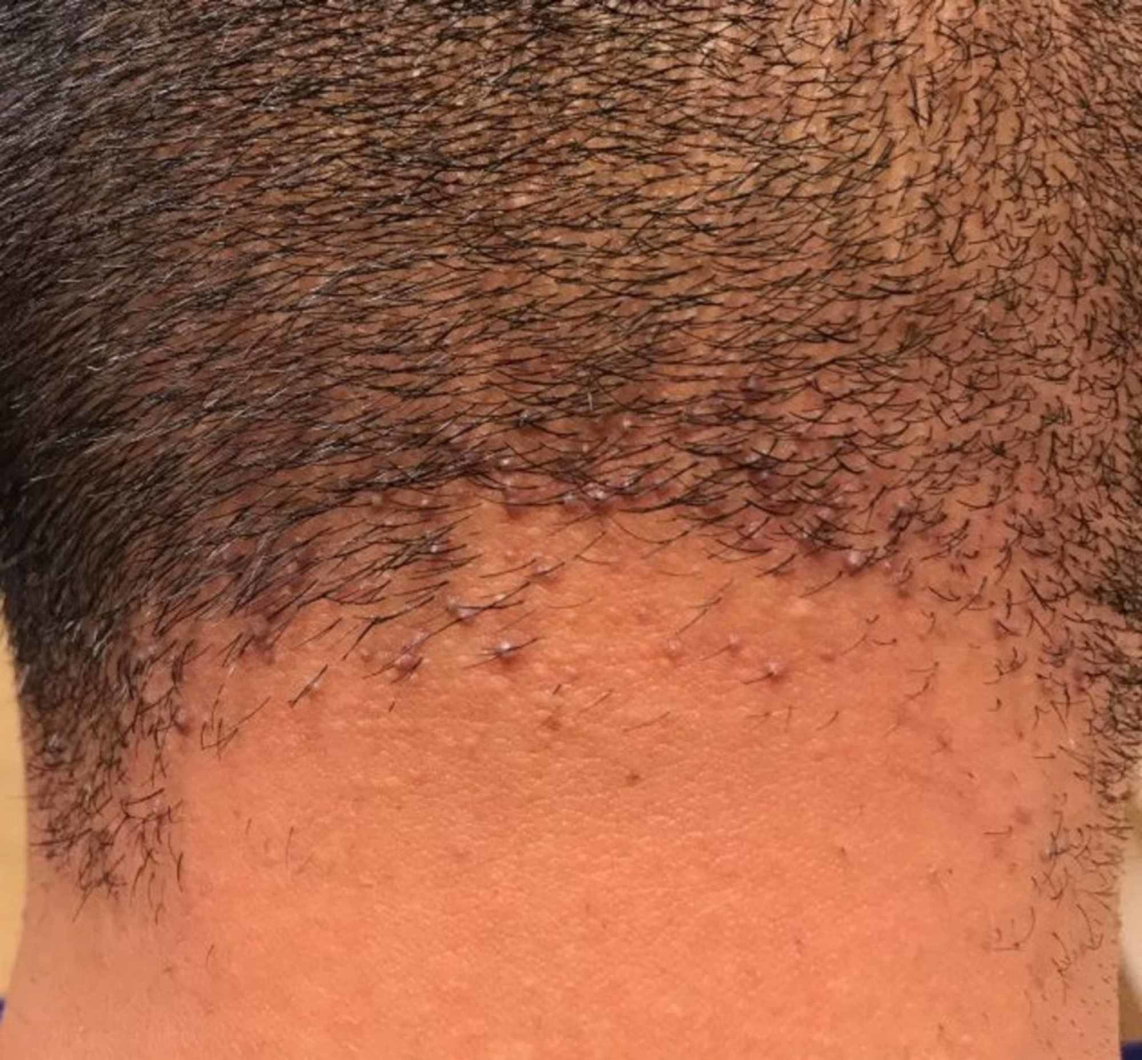
Acne keloidalis nuchae

**Figure 3:**
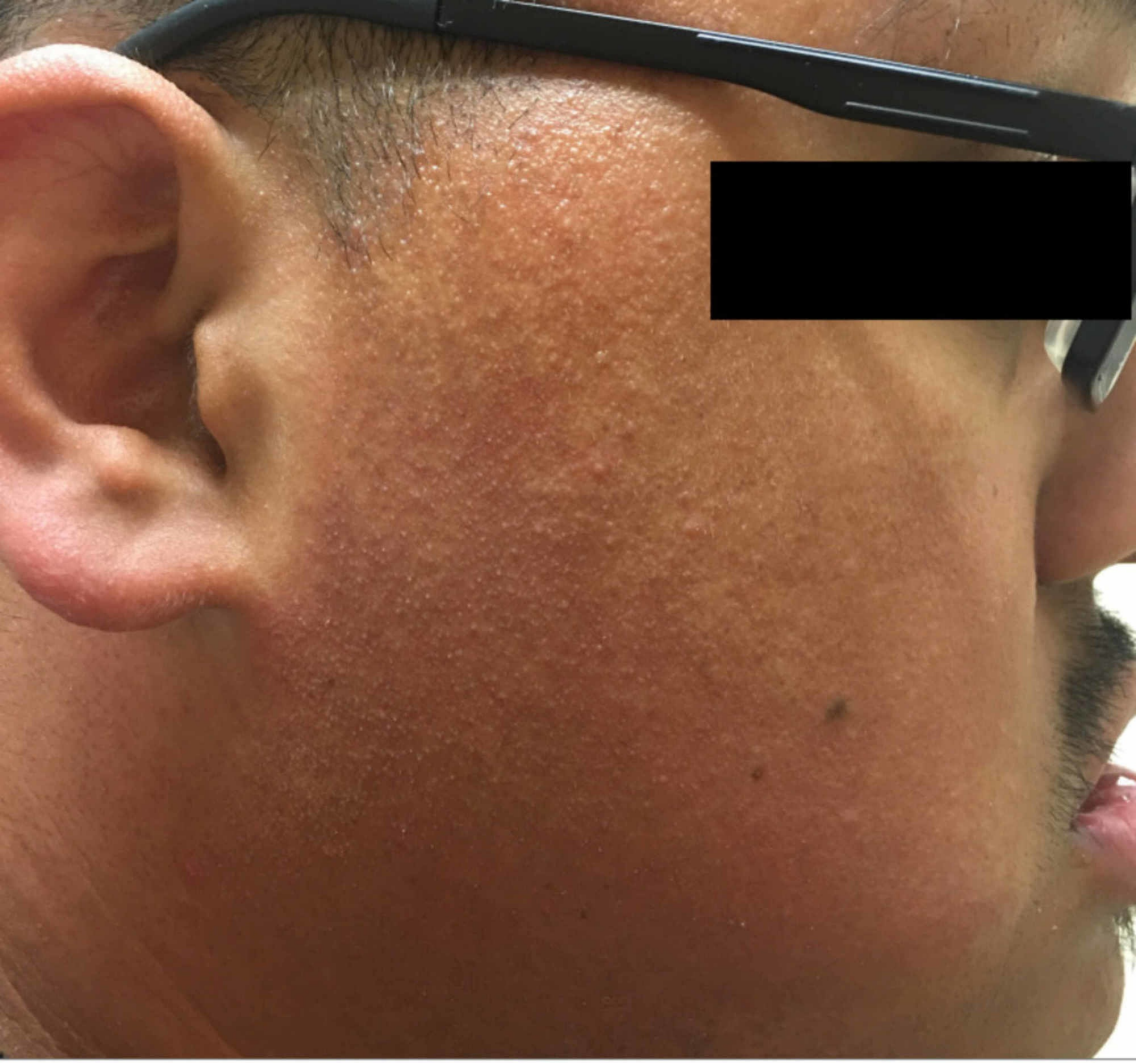
Erythromelanosis follicularis faciei

**Figure 4:**
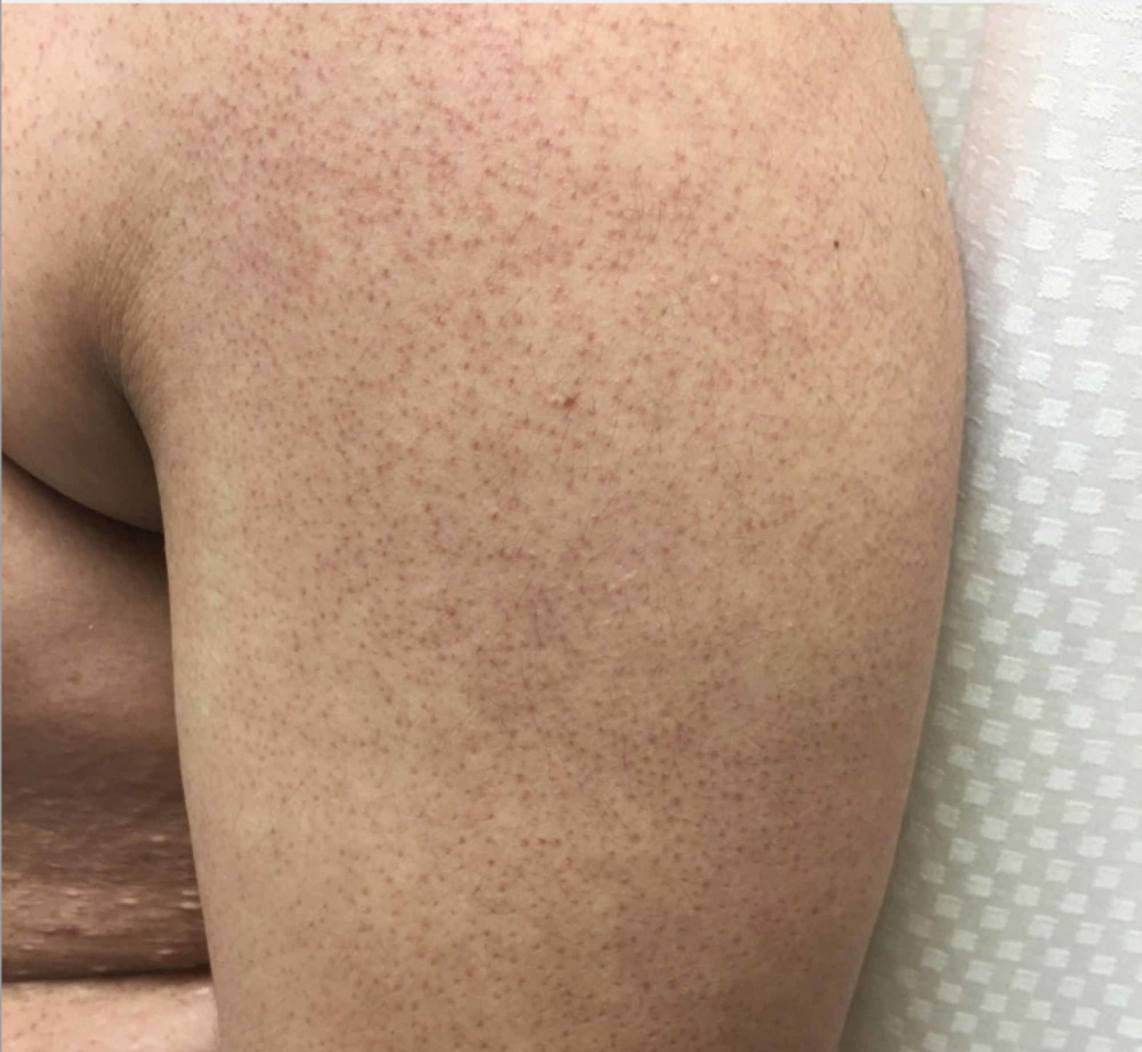
Keratosis pilaris

On physical examination, the patient had scattered erythematous cystic papules in the abdominal fold, on the buttocks, and in the suprapubic region. In the suprapubic region, there were also open comedones and double-headed comedones. He had hyperpigmented scars and nodules in the groin folds (Figure 1). Several firm, dull erythematous follicular based conical papules were appreciated on the posterior neck and occipital scalp (Figure 2). Additional physical exam findings were incidentally noted. On the forehead and cheeks, there were follicular based pinpoint papules on a background of reddish hyperpigmentation (Figure 3). On the extensor arms, trunk, and legs, he had multiple pinpoint erythematous follicular papules (Figure 4).

## Discussion

Hidradenitis suppurativa is a chronic condition characterized by double-headed comedones, recurrent inflamed papules and nodules, and draining sinus tracts with subsequent scarring. Pathogenesis begins with occlusion of the pilosebaceous units, leading to inflammation and rupture of the hair follicle. Keratin and bacteria introduced into the surrounding dermis results in the formation of inflammatory papules and nodules; typical lesions are located in intertriginous zones. Over time, draining sinus tracts and scars may develop [1].

Ours is the first reported case of HS in association with SMS. Interestingly, our patient also had several other follicular-based skin diseases including erythromelanosis follicularis faciei, keratosis pilaris, and acne keloidalis nuchae. SMS is a neurodevelopmental disorder caused by a deletion on chromosome 17 encompassing the retinoic-acid-induced 1 (RAI1) gene. The incidence is 1 in 25,000. The syndrome is characterized by intellectual disability, sleep disturbances, early-onset obesity, and distinct behavioral abnormalities (such as self-injurious behavior) [5]. Guerin-Moreau et al. examined dermatologic findings in 20 patients with SMS. A majority of the patients exhibited features secondary to self-injurious behavior such as onychotillomania, self-biting, and skin picking. Of note, palmoplantar keratoderma, pachydermia, folliculitis of the back, and keratosis pilaris were also observed in a majority of the patients [6].

HS has been associated with other dermatologic diseases, including the follicular occlusion tetrad (acne conglobata, dissecting cellulitis, pilonidal sinus, hidradenitis suppurativa), SAPHO (synovitis, acne, pustulosis, hyperostosis, and osteitis) syndrome, PASH (pyoderma gangrenosum, acne, and suppurativa hidradenitis) syndrome, and PAPASH (pyogenic arthritis, pyoderma gangrenosum, acne, and suppurativa hidradenitis) syndrome [4]. There have also been reports of hidradenitis suppurativa in association with multisystem genetic disorders.

Down Syndrome is one of the most common chromosomal disorders in the United States, resulting from extra genetic material on chromosome 21. A recent large population-based study found a statistically significant association between Down syndrome and HS, with patients with Down syndrome being five times more likely to develop HS when compared to patients without Down syndrome [2]. The mechanism for this association has not been fully elucidated, but it has been suggested that increased expression of amyloid precursor protein stimulates keratinocyte hyperproliferation and follicular occlusion in the epidermis of these patients [2]. Another large population-based study identified a statistically significant association between HS and FMF, an autosomal recessive autoinflammatory disorder characterized by relapsing episodes of fever, arthritis, and peritonitis [7]. Again, the reason for this is unclear but overproduction of pro-inflammatory IL-1β resulting from the mutated MEFV gene implicated in FMF may play a role [7]. Notably, there are reports of both FMF and HS patients responding to pharmacological treatment with IL-1β antagonists [7]. Rare case reports have described a possible association between HS and other multisystem genetic diseases, including KID syndrome, Bazex-Dupre-Christol syndrome, and myotonic dystrophy, with authors suggesting possible predisposing hyperproliferative, occlusive, and hormonal mechanisms for these occurrences [3-4,8-9]. However, these reports present limited observational data and further investigation is necessary to corroborate the proposed associations.

Regarding our case, our patient’s HS improved after three months of doxycycline and topical clindamycin. His acne keloidalis nuchae improved after two months of applying triamcinolone 0.1% cream. He was advised to start a trial of tretinoin 0.025% gel for his erythromelanosis follicularis faciei, and ammonium lactate lotion for his keratosis pilaris. Given the cutaneous findings noted in the study by Guerin-Moreau et al. [6], SMS patients seem to have a tendency toward follicular obstruction and a hyperproliferative epidermis, which may play a role in predisposing them to follicular skin disorders including HS. Similar to Down Syndrome, SMS shares obesity as a comorbidity with HS, which may also play a role in the pathogenesis of HS [2,10-11]. However, this is a limited single observational case report and it is important to consider that other factors, including a possible family history of HS, could have influenced the findings of this case. 

## Conclusions

To our knowledge, this is the first report of HS occurring in a patient with Smith Magenis syndrome. Given the previously described predilection for follicular obstruction in patients with SMS and the presence of several co-existing follicular-based skin diseases in our patient, we suggest the possibility that patients with SMS may have a predisposition for follicular skin disorders, including HS. Further research is necessary to investigate this possible association.
